# A novel endoplasmic reticulum stress-related lncRNA signature for prognosis prediction and immune response evaluation in Stomach adenocarcinoma

**DOI:** 10.1186/s12876-023-03001-0

**Published:** 2023-12-09

**Authors:** Zhaoxiang Song, Mengge Su, Xiangyu Li, Jinlin Xie, Fei Han, Jianning Yao

**Affiliations:** Depratment of Gastroenterology, The First Affiliated Hospital of Zhengzhou University, Zhengzhou, China

**Keywords:** Stomach adenocarcinoma, Endoplasmic reticulum stress, lncRNA, Prognosis, Immune infiltration, Drug therapy

## Abstract

**background:**

Stomach adenocarcinoma (STAD) is a significant contributor to cancer-related mortality worldwide. Although previous research has identified endoplasmic reticulum stress (ERS) as a regulator of various tumor-promoting properties of cancer cells, the impact of ERS-related long non-coding RNAs (lncRNAs) on STAD prognosis has not yet been investigated. Therefore, our study aims to develop and validate an ERS-related lncRNA signature that can accurately predict the prognosis of STAD patients.

**Methods:**

We collected RNA expression profiles and clinical data of STAD patients from The Cancer Genome Atlas (TCGA) and identified ERS-related genes from the Molecular Signature Database (MSigDB). Co-expression analysis enabled us to identify ERS-related lncRNAs, and we applied univariate Cox, least absolute shrinkage, and selection operator (LASSO), and multivariate Cox regression analyses to construct a predictive signature comprising of 9 ERS-related lncRNAs. We assessed the prognostic accuracy of our signature using Kaplan-Meier survival analysis, and validated our predictive signature in an independent gene expression omnibus (GEO) cohort. We also performed tumor mutational burden (TMB) and tumor immune microenvironment (TIME) analyses. Enrichment analysis was used to investigate the functions and biological processes of the signature, and we identified two distinct STAD patient subgroups through consensus clustering. Finally, we performed drug sensitivity analysis and immunologic efficacy analysis to explore further insights.

**Results:**

The 9 ERS related-lncRNAs signature demonstrated satisfactory predictive performance as an independent prognostic marker and was significantly associated with STAD clinicopathological characteristics. Furthermore, patients in the high-risk group displayed a worse STAD prognosis than those in the low-risk group. Notably, gene set enrichment analysis (GSEA) revealed significant enrichment of extracellular matrix pathways in the high-risk group, indicating their involvement in STAD progression. Additionally, the high-risk group exhibited significantly lower TMB expression levels than the low-risk group. Consensus clustering revealed two distinct STAD patient subgroups, with Cluster 1 exhibiting higher immune cell infiltration and more active immune functions. Drug sensitivity analysis suggested that the low-risk group was more responsive to oxaliplatin, epirubicinl, and other drugs.

**Conclusion:**

Our study highlights the crucial regulatory roles of ERS-related lncRNAs in STAD, with significant clinical implications. The 9-lncRNA signature we have constructed represents a reliable prognostic indicator that has the potential to inform more personalized treatment decisions for STAD patients. These findings shed new light on the pathogenesis of STAD and its underlying molecular mechanisms, offering opportunities for novel therapeutic strategies to be developed for STAD patients.

**Supplementary Information:**

The online version contains supplementary material available at 10.1186/s12876-023-03001-0.

## Introduction

Stomach adenocarcinoma (STAD) represents a globally prevalent malignancy, exerting a substantial impact on human health [1]. As the predominant histological subtype of gastrointestinal malignancies, it constitutes 95% of Gastric Cancer (GC) cases [2, 3]. A range of treatment modalities, encompassing systemic chemotherapy, radiotherapy, surgery, immunotherapy, and targeted therapy, have exhibited efficacy in combating gastric adenocarcinoma [4]. Nonetheless, therapeutic outcomes for advanced-stage STAD remain suboptimal [5], with a dismal 5-year overall survival rate below 10% in patients afflicted with advanced STAD [6]. Consequently, the identification of biomarkers, specifically microsatellite instability (MSI), programmed cell death ligand 1 (PD-L1), human epidermal growth factor receptor 2 (HER2), tumor mutation burden (TMB), and Epstein-Barr virus (EBV), has become increasingly crucial in guiding systemic therapy strategies [4]. This underscores the urgency for developing an accurate prognostic model and novel therapeutic targets in the context of STAD.

Long noncoding RNAs (lncRNAs), characterized as RNA transcripts exceeding 200 nucleotides in length, serve multifaceted roles in cellular biology [7]. In an array of cancers, including STAD, dysregulated lncRNAs are implicated in tumor metastasis through the modulation of processes such as epithelial-mesenchymal transition (EMT), vascular metastasis, and tumor colonization [8, 9]. Accumulating evidence suggests that lncRNAs are not only key regulators of cancer pathways but also biomarkers of disease [10, 11]. Within the context of STAD, specific lncRNAs have been identified as contributing factors to disease development and progression. For instance, the lncRNA GMAN is upregulated in gastric cancer tissues, facilitating metastasis via binding to GMAN-AS and augmenting the translation of Ephrin A [12]. In a parallel manner, lncRNA GClnc1 has been demonstrated to promote gastric carcinogenesis by functioning as a modular scaffold for WDR5 and KAT2A complexes, thereby determining histone modification patterns [13].

Endoplasmic reticulum stress (ERS) is the response to some physiological and pathological injuries, such as nutrient fluctuations, increased demands on protein secretion, hypoxia, mutations in client proteins of the secretory pathway that stabilize or promote aggregation of intermediate folding forms, and reduction in calcium levels with inhibitory effects on calcium-dependent haperones, the capacity to either properly fold proteins or dispose of those that fail quality control is overwhelmed, the ER lumen will begin to accumulate misfolded proteins, threatening the function and survival of cell [14–17]. ERS elicits a cascade of signal transduction pathways and regulatory mechanisms aimed at restoring ER protein homeostasis, an adaptive response referred to as the unfolded protein response (UPR) [18]. The UPR transduces information about the protein-folding status in the ER lumen to the nucleus and cytosol to buffer fluctuations in unfolded protein load [19, 20]. When cells undergo irreversible ERS, this pathway eliminates damaged cells by apoptosis [21]. The burgeoning role of UPR has been observed in various aspects of tumorigenesis, encompassing angiogenesis, tumor growth, invasion, metastasis, immune evasion, and resistance to chemotherapy and radiotherapy [22]. These observations suggest that ERS may constitute a potential therapeutic target in the treatment of malignant tumors.

However, the relationship between STAD and ERS-related lncRNAs remains ambiguous. In this study, our objective is to establish a predictive signature predicated on ERS-related lncRNAs, and to assess its implications for prognosis, diagnosis, tumor mutation burden (TMB), and drug sensitivity in STAD patients. Moreover, we will scrutinize the function, biological pathway, and tumor immune microenvironment (TIME) to elucidate the underlying mechanisms of ERS in STAD. These findings will not only lay the groundwork for further investigation into ERS molecules and mechanisms but also facilitate the advancement of targeted therapy for STAD.

## Materials and methods

### Data acquisition

The RNA sequencing data, consisting of 379 tumor samples and 34 normal samples, and clinical data from 406 samples, were sourced from The Cancer Genome Atlas (TCGA https://portal.gdc.cancer.gov/). The gene expression profile matrixes of the STAD cohort from GSE26901 were downloaded from the GEO website (https://www.ncbi.nlm.nih.gov/geo/) for validation. The molecular signature database (MSigDB http://www.gsea-msigdb.org/gsea/msigdb/index.jsp) was searched using the keywords "endoplasmic reticulum stress" to obtain 330 ERS-related genes for analysis. We obtained resources of 29 immune signatures (16 immune cells and 13 immune functions) and 47 immune checkpoint genes from Additional file 1 of the article by He Yin et al. [23].

### Identification of ERS-related lncRNAs

Gene expression matrices were constructed using the “limma” R package. The Pearson |correlation coefficient|> 0.6, *p*-value < 0.001 were viewed as the standards to identify ERS-related lncRNAs. With the standard of |log2 fold change (FC) |> 1 and the false discovery rate (FDR) < 0.05, we screened 674 ERS-related differentially expressed lncRNAs for further analysis by running the “limma” R package, and generated volcano plot and heatmap for visualization using the “pheatmap” R package.

### Establishment and verification of ERS-related prognostic signature

To obtain the ERS-related lncRNAs associated with survival, we performed univariate Cox regression analysis on the lncRNAs obtained in the previous step. To prevent over-fitting of the prognostic signature, we used Least Absolute Shrinkage and Selection Operator (LASSO) regression analysis for further screening. Finally, we established an ERS-related lncRNA prognostic model using multivariate Cox regression analysis. We employed various R packages, including “survival”, “caret”, “glmnet”, “survminer”, “timeROC”, and “pheatmap”, to perform these analyses.

$$Riskscore=\sum\limits_{i=1}^{n}coef\left(i\right)\times \mathrm{exp}(i)$$

The relative expression level of each ERS-related lncRNA was denoted as Exp(i), and the regression coefficient of each lncRNA was denoted as Coef(i), which was computed by multivariate Cox regression analysis. Using the median value of the risk score as a cutoff, we divided STAD patients into high-risk and low-risk groups.

To evaluate the prognostic value of our model, we employed the "survival" and “survminer” R packages to generate Kaplan–Meier survival curves and compared the survival differences between the high-risk and low-risk groups. We randomly grouped 343 STAD patients with complete clinical information into training set: test set at a ratio of 1:1, and subsequently conducted internal validation of the prognostic model. We also used the “pheatmap” R package to produce risk curves, survival status maps, and risk heatmaps for the complete, training, and test sets. In addition, we generated receiver operating characteristic (ROC) curves and calculated the area under the curve (AUC) using the “survival”, “survminer” and “timeROC” R packages. To further explore the clinical relevance of our prognostic signature, we plotted Kaplan–Meier survival curves to investigate the association between clinicopathological characteristics and overall survival (OS) in the high-risk and low-risk groups.

### Evaluation of the prognostic value of ERS-related lncRNAs in an independent GEO validation cohort

The STAD patients in GEO were grouped into low and high expression subgroups based on the median level of the lncRNAs [24]. The Kaplan‒Meier method using the R “survival” package was further conducted to compare the OS between the two subgroups, and then the “survival” and “survminer” packages were used to plot the survival curves.

### Establishment of nomogram

We combined the riskscore with several clinicopathological features (age, sex, stage, stage), with the “regplot”, “survival” and “rms” R packages, to establish a nomogram that can predict the 1 -, 3 -, and 5-year survival probability of STAD patients. The calibration curve was drawn to test the accuracy of the nomogram of clinical prognosis.

### Enrichment analysis

Based on log2 FC > 1 and FDR < 0.05 as screening criteria, we obtained differentially expressed genes in high-risk and low-risk groups. The gene ontology (GO) and Kyoto Encyclopedia of Genes and Genomes (KEGG) enrichment analysis were performed to find the enriched biological pathways and functions related to the ERS-related genes by “clusterprofiler” R package. The enriched results for GO and KEGG analysis were visualized by the “ggplot2” R package. Through gene set enrichment analysis (GSEA), we identified these biological pathways associated with the ERS-related lncRNAs.

### TMB analysis

Mutation data were downloaded from the cbioportal database, and the “maftools” R package was used to visualize somatic mutation data in the mutation annotation format. The TMB value was then calculated by adding the number of somatic mutations to the length of the exon. The differences in TMB values between the high-risk and low-risk groups were analyzed. Additionally, the KM survival curve was drawn to analyze the risk score and TMB of STAD patients survival.

### Estimation of the tumor immune microenvironment, immune cell infiltration and immune checkpoint molecules

To assess tumor immunoactivity, we estimated the abundance of stromal cells and immune cells based on ESTIMATE data, a method that calculates the proportion of immune and stromal cells in each tumor sample, thereby quantifying tumor purity (level of immune cell invasion) based on the expression of immune genes. In detail, we performed the ESTIMATE algorithm in R language to estimate the proportion of immune or stromal components in the TME, where we got the following scores: ImmuneScore, StromalScore, and ESTIMATEScore, which are positively associated with the ratio of immune components, stromal components, and the sum of both components. The higher the scores are, the larger the ratio of the corresponding components in the TME. Then, so as to further explore the differences in the tumor immune microenvironment between the high-risk and the low-risk groups, we calculated the proportion of infiltrating immune cells in the STAD samples on the basis of the CIBERSORT algorithm [25]. Along with the application of various algorithms which included TIMER [26], CIBERSORT [27], CIBERSORT-ABS [28], QUANTISEQ [29], MCPCOUNTER [30], XCELL [31] and EPIC [32], we also explored the correlation between immune cells and risk scores, and the result was displayed in a bubble chart. Furthermore, we analyzed the expression of 47 immune checkpoint inhibitor (ICI)-related immunosuppressive molecules in different groups.

### Consensus cluster analysis of ERS-related lncRNAs

Based on the expression of 9 ERS-related lncRNAs, we used “ConsensusClusterPlus” R package to classify the tumor types of STAD patients in TCGA database. The cumulative distribution function (CDF) and consensus matrix were utilized to estimate the best number of clusters.

### Drug sensitivity analysis

We downloaded the cell lines and compounds data from Genomics of Drug Sensitivity in Cancer (GDSC) and used the “oncoPredict” R package to score the drug sensitivity of each patient of STAD in TCGA. A boxplot was drawn to compare the differences in drug sensitivity between high-risk and low-risk groups and between tumor cluster 1 and 2.

### Statistical analysis

R software (version 4.2.2) and Strawberry Perl (version 5.3.1) were used to perform all statistical analyses. *P* < 0.05 was regarded as statistical significance.

## Results

### Screening and identification of ERS-related lncRNAs

**Fig. 1:**
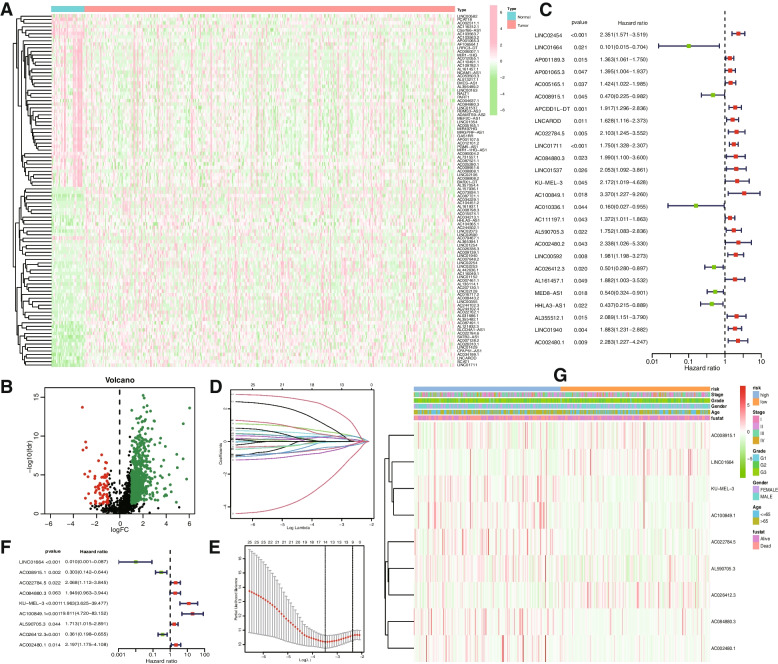
Identification of the prognostic ERS-related lncRNAs for STAD patients. **A** Heatmap of the first 100 differentially expressed lncRNAs. **B** Volcano plot of ERS-related lncRNAs. Green dots indicate upregulated lncRNAs in tumor tissues while the red dots indicate downregulated lncRNAs. **C** Forest plot of multivariate Cox regression analysis. LASSO variable trajectory plot for 1,000 cross validations (**D**) and LASSO coefficient profile **E**. **F** Forest plot of univariate Cox regression analysis. **G** Heatmap showed the expression level of 9 ERS-related lncRNAs and the distribution of clinicopathological features in risk subgroups

We obtained RNA transcription data from 413 samples and clinical information from 406 samples via the TCGA database, subsequently eliminating instances with unknown clinical information for further analysis. Employing 330 ERS-related genes as a basis, we identified 1,620 ERS-related lncRNAs using the Pearson correlation algorithm. From these, we isolated 674 differentially expressed lncRNAs in tumor and normal tissues for subsequent univariate Cox regression analysis. The heatmap in Fig. 1A illustrates the top 100 ERS-related lncRNAs expressed in both normal and tumor tissues. As shown in the volcano plot (Fig. 1B), 609 lncRNAs were upregulated, whereas 65 lncRNAs were downregulated in STAD tissue.

### Establishment and verification of ERS-related prognostic signature

Utilizing univariate Cox regression analysis, we identified 26 ERS-related lncRNAs with prognostic value in STAD patients and generated a forest plot (Fig. 1C). Subsequently, we employed LASSO regression to filter the lncRNAs and conducted cross-validation (Fig. 1D, E). Ultimately, multivariate Cox regression analysis was performed to establish the risk model. As a result, the nine most informative lncRNAs (LINC01664, AC008915.1, AC022784.5, AC084880.3, KU-MEL-3, AC100849.1, AL590705.3, AC026412.3, AC002480.1) were selected as prognostic markers for signature construction. We then created a forest plot and heatmap for the prognostic nine ERS-related lncRNAs (Fig. 1F, G).

**Fig. 2:**
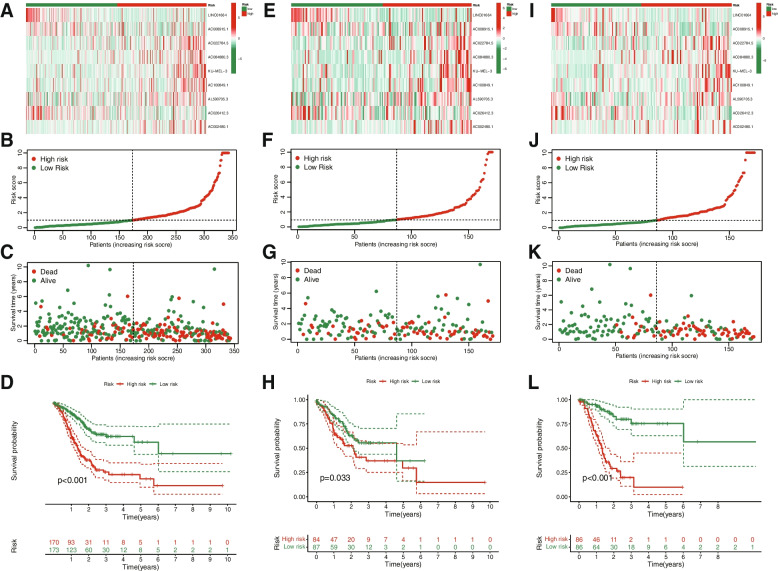
Prognosis and risk scoring analysis of 9 ERS-related lncRNAs in different sets for STAD patients. Heatmaps of 9 ERS-related lncRNAs expression in high-and low-risk groups, scatterplots of survival status and risk scores of STAD patients, and KM survival curves in the complete set (**A**-**D**), test set (**E**–**H**), and training set **I**-**L**

Based on the median risk score, STAD patients were divided into low-risk and high-risk groups. We generated heatmaps depicting the expression of the nine ERS-related lncRNAs in high- and low-risk groups, scatterplots illustrating the survival status and risk scores of STAD patients, and Kaplan–Meier survival curves for the complete set (Fig. 2A-D), test set (Fig. 2E-H), and training set (Fig. 2I-L). The survival rate of high-risk STAD patients was notably lower than that of low-risk patients. The risk score ranking distributions and scatterplots revealed a correlation between the survival status of STAD patients and the risk score, indicating that as the risk score increased, so did the mortality rate of patients. These results suggest that the newly constructed risk model serves as a superior prognostic predictor.

**Fig. 3:**
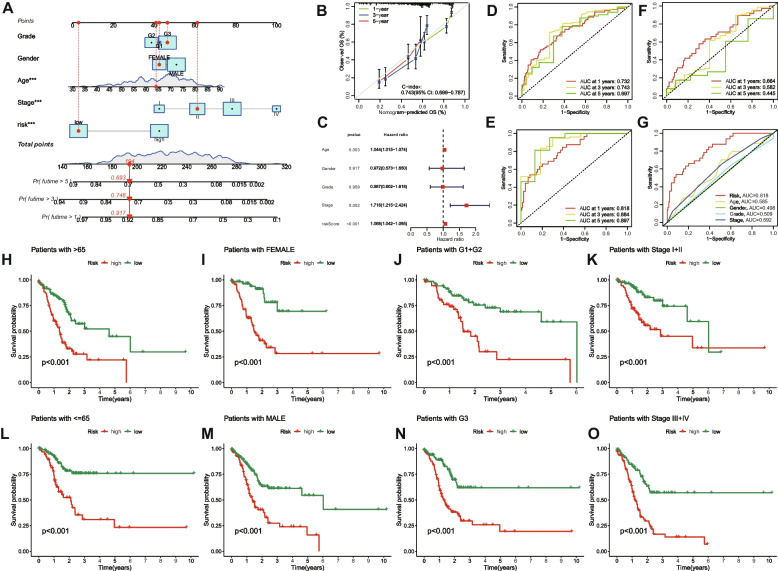
Validation of the ERS-related lncRNA prognostic signature and its relationship with clinicopathological characteristics. A nomogram integrating grade, gender, age, stage and risk to predict 1-, 3-, and 5-year OS for STAD patients **A**. Calibration curves for the relationship between predicted survival and the observed OS rate at 1, 3, and 5 years **B**. Forest plot of multivariate Cox regression **C**. ROC curves and AUCs for 1-, 3-, and 5-year survival rates of the complete set, training set and test set **D**, **E** and **F**. ROC curves and AUCs of the risk score and clinicopathological characteristics **G**. Kaplan–Meier survival curves of STAD patients’ OS on the clinicopathological characteristics between two groups in the complete set **H**–**O**

To determine whether prognostic markers can serve as independent prognostic indicators in patients with STAD, univariate and multivariate Cox regression analyses were conducted. The results demonstrated that age, stage, and risk score function as independent prognostic factors in STAD patients. Concurrently, to predict the 1-, 3-, and 5-year OS of STAD patients, we incorporated age, sex, stage, grade, and risk score to develop a nomogram (Fig. 3A) and generated a calibration curve (Fig. 3B) to validate the nomogram's accuracy. The forest plot of multivariate Cox regression was subsequently produced based on risk scores and clinicopathological features (Fig. 3C).

The AUC values predicted for 1-, 3-, and 5-year OS were 0.732, 0.743, and 0.697 in the complete set (Fig. 3D), 0.818, 0.884, and 0.897 in the training set (Fig. 3E), and 0.664, 0.582, and 0.445 in the test set (Fig. 3F). Figure 3G revealed that the AUC value of the risk score surpassed other clinicopathological characteristics, indicating superior predictive performance. Regarding the correlation between clinicopathological features and the prognostic model, Fig. 3H-O demonstrated that STAD patients in the high-risk and low-risk groups with distinct clinicopathological characteristics exhibited significant differences in OS (all *P* < 0.001).

### Evaluation of the prognostic value of ERS-related lncRNAs in an independent gene expression omnibus (GEO) validation cohort

**Fig. 4:**
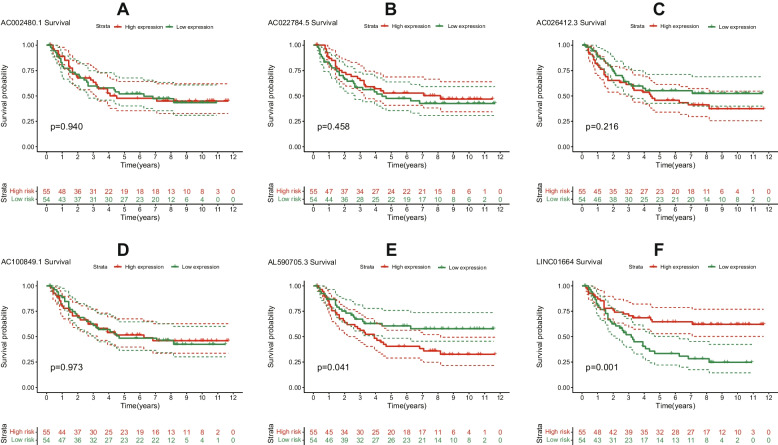
Evaluation of the prognostic value of ERS-related lncRNAs in an independent GEO validation cohort. K-M survival curves for 6 target lncRNAs in STAD **A**-**F**

To validate the prognostic value of the 9 ERS-related lncRNAs, the GEO validation cohorts were divided into high- and low-expression groups. Due to the small number of STAD samples in the GEO dataset, 3 lncRNAs (AC008915.1, AC084880.3 and KU-MEL-3) were not expressed in the GEO dataset, so we grouped the sample based on the median expression of the remaining 6 lncRNAs. The survival rate of STAD patients was statistically signifcant between LINC01664 and AL590705.3 of the high- and low-expression groups (*P* < 0.05) (Fig. 4).

### Enrichment analysis

**Fig. 5:**
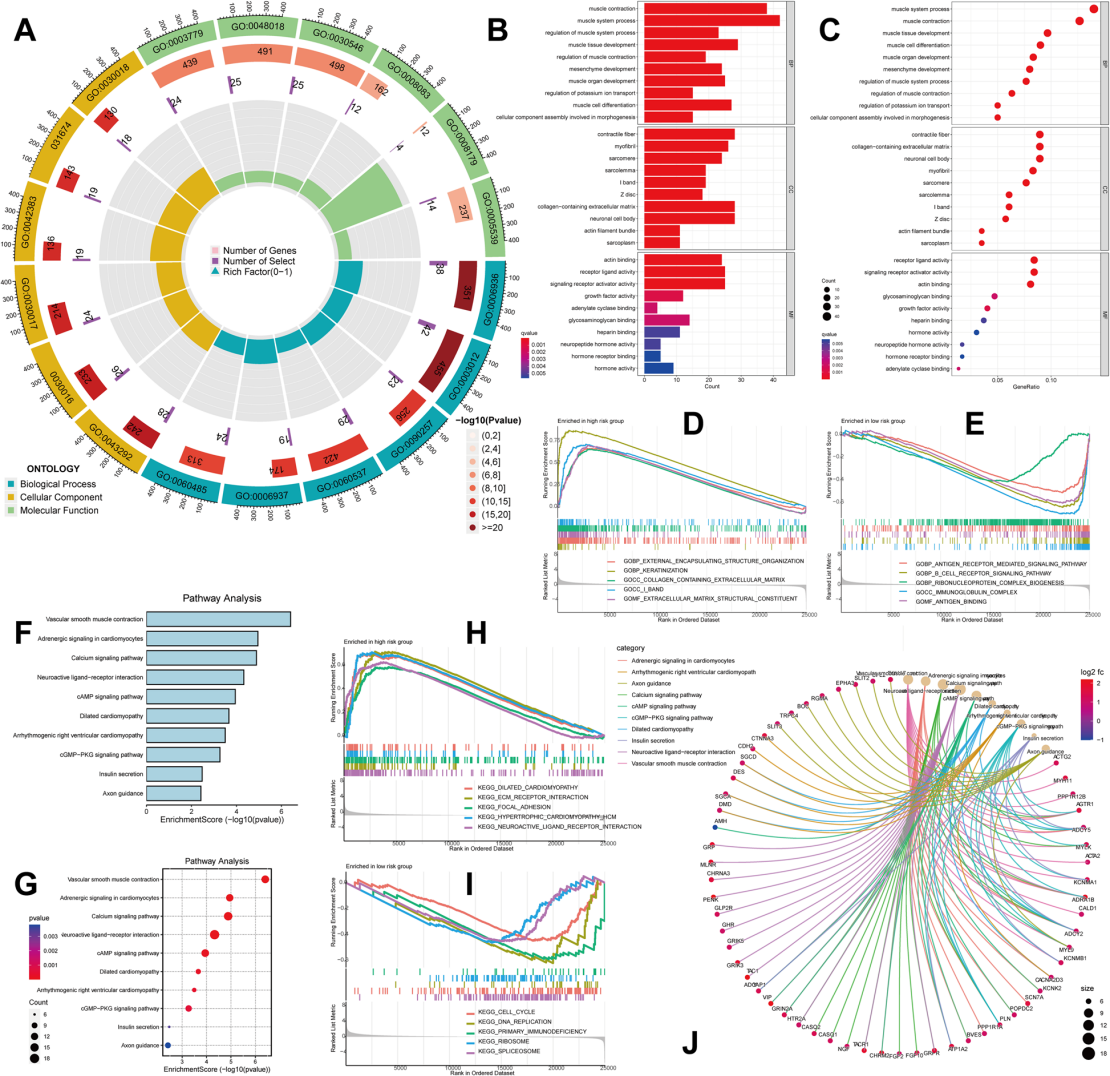
Enrichment analysis of the prognostic signature. The circle plot of GO analysis **A**. The GO function enrichment analyses **B**, **C**. GSEA analysis of GO pathways **D**, **E**. The KEGG function enrichment analyses [33, 34] **F**, **G**. GSEA analysis of KEGG pathways **H**, **I**. The circle plot of KEGG analysis **J**

We identified 364 ERS-related differentially expressed genes (DEGs) in high-risk and low-risk groups, comprising 358 upregulated genes and 6 downregulated genes. To investigate the biological function of ERS-related genes, we conducted GO, KEGG enrichment analysis, and GSEA analysis. GO enrichment analysis indicated that ERS-related genes were predominantly enriched in collagen-containing extracellular matrix, contractile fiber, receptor ligand activity, and muscle system process (Fig. 5A-C). For GSEA analysis, we selected the GO database, and the results showed that external encapsulating structure organization, keratinization, and collagen-containing extracellular matrix were primarily enriched in the high-risk groups (Fig. 5D), while B cell receptor signaling pathway, antigen receptor-mediated signaling pathway, and immunoglobulin complex were chiefly enriched in the low-risk groups (Fig. 5E). KEGG enrichment analysis revealed that the enriched genes were mainly involved in vascular smooth muscle contraction, adrenergic signaling in cardiomyocytes, calcium signaling pathway, and neuroactive ligand-receptor interaction (Fig. 5F, G). We selected the KEGG database for GSEA analysis, and the results demonstrated that dilated cardiomyopathy, ECM receptor interaction, and focal adhesion were predominantly enriched in the high-risk groups (Fig. 5H), while cell cycle, DNA replication, and primary immunodeficiency were mainly enriched in the low-risk groups 5I). Ultimately, we generated a KEGG circle plot to illustrate the relationships between different pathways (Fig. 5J).

### TMB analysis

**Fig. 6:**
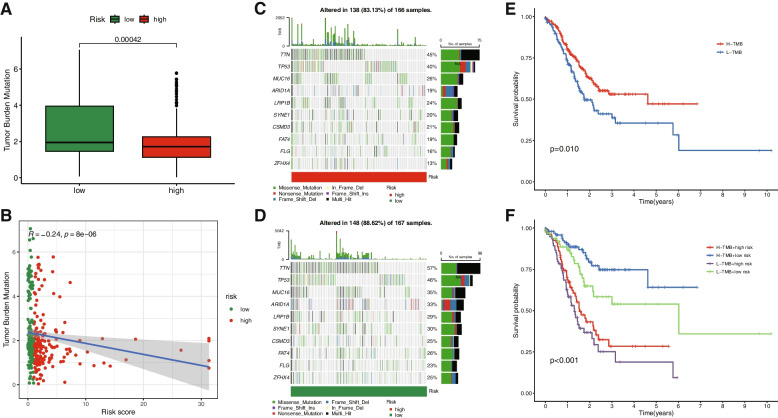
TMB analysis of the prognostic signature. The bean plot for the differences in TMB between high- and low-risk groups **A**. The correlation curve between TMB and the risk score **B**. The waterfall plots of the tumor mutation rate in the high-risk group and low-risk group based on the prognostic signature **C**, **D**. Kaplan–Meier survival curves of BC patients between the H-TMB and L-TMB groups **E**. Kaplan–Meier survival curves of BC patients across H-TMB + high risk, H-TMB + low risk, L-TMB + high risk, and L-TMB + low risk **F**

Based on the risk score, we employed the R package "maftools" to analyze the gene mutation profile of STAD patients, encompassing a total of 343 STAD samples. TMB in the high-risk group was lower than that in the low-risk group. A significant negative correlation was observed between TMB and risk score (Fig. 6A, B). We conducted a visual analysis of the TMB data for patients with STAD. The waterfall plot revealed that the main mutant genes in both high-risk and low-risk groups included TTN, TP53, MUC16, ARID1A, LRP1B, SYNE1, CSMD3, FAT4, FLG, and ZFHX4 (Fig. 6C, D). The survival curve demonstrated that patients with high TMB had a significantly longer survival time than those with low TMB, and the prognosis was most favorable for patients with high TMB and low risk (Fig. 6E, F).

### Estimation of the tumor immune microenvironment, immune cell infiltration and immune-related functional

**Fig. 7:**
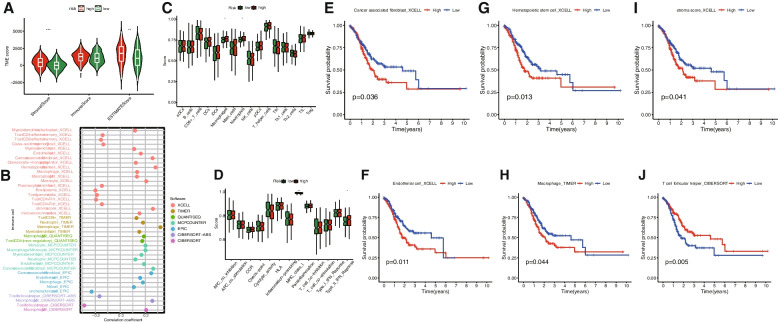
Exploration of the tumor immune status. **A** The violin plot for StromalScore, ImmuneScore, and ESTIMATEScore in the high- and low-risk groups. **B** Estimation of immune-infiltrating cells in STAD. The score of the infiltrating immune cells (**J**) and immune-related functions (**K**) in the high- and low-risk groups. Kaplan–Meier survival curves of Cancer associated fibroblast (**E**), Endothelial cell (**F**), Hematopoietic stem cell (**G**), Macrophage (**H**), stroma score (**I**) and T cell follicular helper (**J**) in STAD patients. *, *p* < 0.05; **, *p* < 0.01; ***, *p* < 0.001

The violin plot (Fig. 7A) revealed that the ImmuneScore, StromalScore, and ESTIMATEScore in the high-risk group were higher than those in the low-risk group and there was significant difference between the latter two groups (*P* = 0.19, 6.5e-07, 0.0011). Immune correlation analysis (Fig. 7B) demonstrated that most immune cells were positively correlated with the patients' risk scores. Myeloid dendritic cells, hematopoietic stem cells, cancer-associated fibroblasts, and stroma scores exhibited higher correlations. In contrast, uncharacterized cells and T cell follicular helper cells were negatively correlated with the risk score. In addition to the ssGSEA condensed score, we further investigated the relationship between risk score and various immune cell subsets and functions. In high-risk patients, almost all immune-related functional cells exhibited higher ssGSEA scores, excluding aDCs, Tfh, and Th2 cells, and there was a significant difference between high-risk and low-risk groups in Macrophages and Neutrophils (Fig. 7C). The results showed that except for MHC_class_I, Cytolytic_activity, Inflammation-promote and T_cell_co-stimulation, the immune function score of the high risk group was higher than that of the low risk group, and there was significant difference in Type_II_IFN_Reponse. (Fig. 7D). Consequently, patients in the high-risk group demonstrated stronger immune cell activity and immune function. Furthermore, survival analysis indicated that the survival time of patients in the low-risk group was significantly longer than that of the high-risk group for hematopoietic stem cells, stroma scores, macrophages, endothelial cells, and cancer-associated fibroblasts (Fig. 7E-I). In contrast, for T cell follicular helper cells, the survival time of patients in the high-risk group was significantly longer than that of the low-risk group (Fig. 7J). These findings are consistent with the results of the immune correlation analysis.

### Consensus cluster of ERS-related lncRNAs identified two clusters of STAD patients

**Fig. 8:**
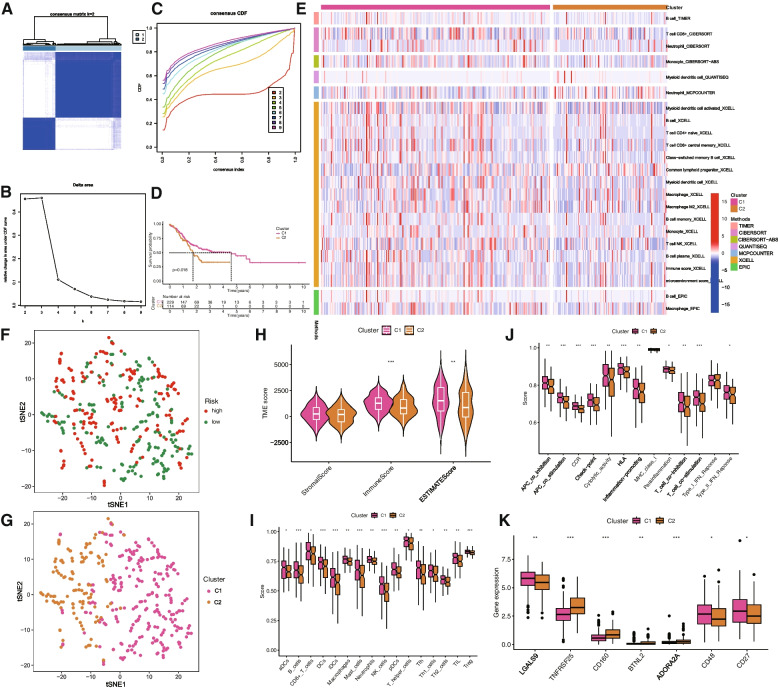
Survival, T-distributed stochastic neighbor embedding (t-SNE), and TIME analysis in consensus clustering subgroups of STAD. **A** The consensus score matrix of all samples when k = 2. **B** Of the length and slope of the CDF curve as the index changes from 2 to 9. **C** Consensus clustering CDF for k = 2 to 9. **D** K-M curves showed differences in OS between Cluster1 and Cluster2. **E** Heatmap of immune-infiltrating cells in two subgroups. **F** t-SNE analysis between the high- and low-risk groups. **G** t-SNE analysis between Cluster1 and Cluster2. **H** The violin plot for StromalScore, ImmuneScore, and ESTIMATEScore in Cluster1 and Cluster2. The score of the infiltrating immune cells (**I**) and immune-related functions (**J**) in Cluster1 and Cluster2. (**K**) The boxplot shows the expression of 7 immune checkpoint molecules between the two subgroups

To further explore the molecular subtypes of STAD, we subjected nine ERSLs to consensus cluster analysis. Based on the results of the CDF and clustering matrix, we determined that k = 2 was a more suitable number of subtypes (Fig. 8A-C). STAD samples were divided into C1 (*n* = 229) and C2 (*n* = 114). The KM survival curve (Fig. 8D) demonstrated that patients in group C1 exhibited longer survival times than those in group C2. Consistent with the immune score, group C1 exhibited greater immune cell infiltration than group C2, as illustrated in the immune cell infiltration heatmap (Fig. 8E). T-distributed stochastic neighbor embedding (t-SNE) analysis revealed significant differences between distinct tumor subtypes, which were more pronounced than those between the high-risk and low-risk groups (Fig. 8F, G). We then further evaluated the immune status of the two tumor subtype groups, employing the ESTIMATE algorithm to score the patients' TIMEs. The results indicated that patients in group C1 had higher ESTIMATE, Stromal, and Immune scores compared to those in group C2 (Fig. 8H). There were significant differences in immune cell infiltration and immune function among different tumor subtypes (Fig. 8I, J). Group C1 had higher ssGSEA scores, with the exceptions of MHC_class_I and Type_I_IFN_Response. To evaluate the efficacy of immunotherapy in STAD, we further investigated the differences in the expression of immune checkpoints between different subtypes. The results showed that LGALS9, CD48, and CD27 had the highest expression in Cluster 1, while TNFRSF25, CD160, BTNL2, and ADORA2A exhibited the highest expression in Cluster 2 (Fig. 8K).

### Clinical drug sensitivity analysis and immunotherapy efficacy evaluation of the ERS-related lncRNA prognostic signature

**Fig. 9:**
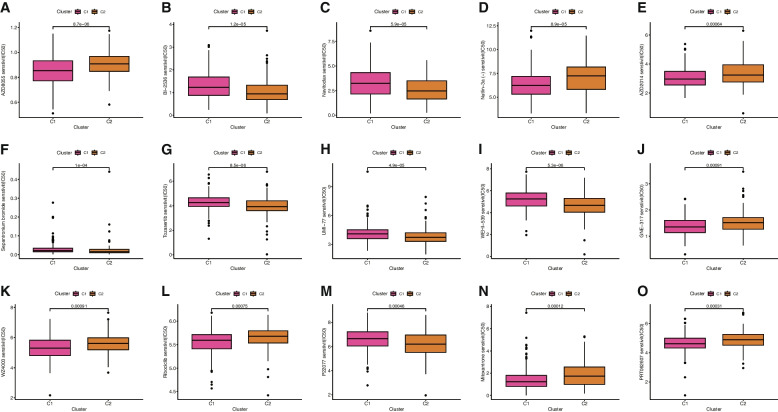
Drug sensitivity correlated with Cluster1 and Cluster2 in STAD as assessed by IC50. **A** AZD8055. **B** BI-2536. **C** Navitoclax. **D** Nutlin-3a (-). **E** AZD2014. **F** Sepantronium bromide. **G** Tozasertib. **H** UMI-77. **I** WEHI-539. **J** GNE-317. **K** WZ4003. **L** Ribociclib. **M** P22077. **N** Mitoxantrone. **O** PRT062607

**Fig. 10:**
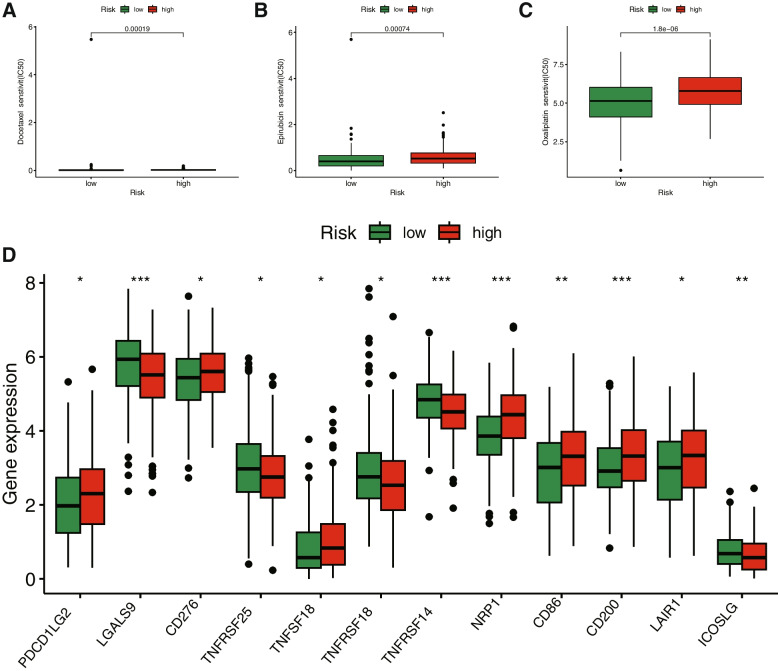
Potential drug sensitivity analysis and the immune checkpoint analysis between the high- and low-risk groups. The boxplots for the drug sensitivity analysis of (**A**) Docetaxel, (**B**) Epirubicin and (**C**) Oxaliplatin. Drugs that have been proven to be effective for gastric cancer were displayed. **D** Differential expression analysis of the immune checkpoint genes between the high- and low-risk groups

The drug treatment of STAD has garnered considerable attention due to its vast research potential. Through a comparative assessment of drug sensitivity using IC50 values, Fig. 9A-O displays 15 drugs with significantly different sensitivities between Cluster 1 and Cluster 2. Notably, STAD patients in Cluster 1 exhibited greater sensitivity to AZD2014, AZD8055, GNE-317, Mitoxantrone, Nutlin-3a (-), PRT062607, Ribociclib, and WZ4003, while those in Cluster 2 were more sensitive to BI-2536, Navitoclax, P22077, Sepantronium bromide, Tozasertib, UMI-77, and WEHI-539. The relationship between risk score and drug resistance was also analyzed and We only showed drugs that have been proved effective in clinical trials for gastric cancer. We observed that Docetaxel displayed higher IC50 values in low-risk patients, while Epirubicin, Oxaliplatin exhibited higher IC50 values in high-risk patients (Fig. 10A-C). As shown in the Additional file 3, compared with the high-risk group, patients in the low-risk group were more sensitive to most antineoplastic drugs, except docetaxel, dasatinib and SB505124. Considering the clinical application and benefits of ICIS, we further investigated the expression of immune checkpoint genes between the high-risk and low-risk groups. Consequently, the expression of PDCD1LG2, CD276, TNFSF18, NRP1, CD86, CD200, and LAIR1 in the high-risk group was higher than in the low-risk group. In contrast, the expression of LGALS9, TNFRSF25, TNFRSF18, TNFRSF14, and ICOSLG in the low-risk group was significantly higher than in the high-risk group (Fig. 10D).

## Discussion

In recent years, substantial advancements have been made in the realm of research aimed at enhancing the prognosis of patients with STAD. As a result, neoadjuvant chemotherapy, radiotherapy, and molecular-targeted therapies have emerged as efficacious approaches [35]. Consequently, the identification of robust molecular biomarkers is of paramount importance for refining the prognostic prediction and evaluation in patients with STAD.

lncRNAs have assumed a pivotal role within the intricate regulatory networks of ERS, exerting significant influence on diverse aspects of human malignancies. One illustrative example involves the transcription factor C/EBP homologous protein (CHOP), which has been firmly established as a central transcriptional regulator responsible for governing the expression of lnc-MGC [36]. Furthermore, the pseudogene Golgin A2 pseudogene 10 (GOLGA2P10) exhibits aberrant expression patterns, leading to an elevation in the expression of the anti-apoptotic gene BCL-xL. This molecular alteration confers upon cancer cells a heightened resistance to the cytotoxic effects of ERS, enabling their survival amidst challenging conditions. Additionally, certain lncRNAs, such as metastasis-associated lung adenocarcinoma transcript 1 (MALAT1), experience upregulation in response to pharmacological agents that induce ERS [37]. Subsequent investigations have unveiled that the activated IRE1 and PERK signaling pathways enhance the expression of MALAT1, subsequently fostering colorectal cancer (CRC) cell migration. Furthermore, the ERS orchestrates the modulation of long non-coding RNA (lncRNA) levels within the context of tumorigenesis. Numerous lncRNAs, known to be associated with tumorigenesis, have been demonstrated to modulate the proliferation and apoptosis of tumor cells by activating the UPR. Recent studies have reported that the ectopic expression of MEG3 culminates in an upregulation of ERS-related proteins, including GRP78, IRE1, PERK, ATF6, and CHOP, concomitant with the nuclear translocation of NF-κB. Consequently, MEG3 is implicated in the inhibition of cancer cell growth and the induction of apoptosis [38–40]. Furthermore, lncRNAs assume a pivotal role in orchestrating these intricate processes. One such example is the Non-coding RNA Activated by DNA Damage (NORAD), which has been extensively documented to display elevated expression levels in various cancer tissues. NORAD actively engages in a multitude of biological processes within the tumor microenvironment, with a notable impact on cellular migration and invasion [41, 42].

Our investigation successfully developed a prognostic model for STAD based on 9 ERS-related lncRNAs, thereby underscoring the potential clinical applicability of ERS-related lncRNAs in STAD. Among these nine lncRNAs, only AC008915.1 has been identified as a prognostic factor in early-stage lung squamous cell carcinoma [43], while AL590705.3 has been reported as a prognostic biomarker in both hepatocellular carcinoma (HCC) and STAD [44, 45]. Additionally, AC026412.3 is considered a prognostic factor for HCC [46], whereas the remaining lncRNAs have not been reported in any cancer studies. In summary, as a novel prognostic risk model, the current understanding of these nine ERS-related lncRNAs remains limited, warranting further investigation in the future.

GSEA revealed that the immunoglobulin complex was highly expressed in the low-risk group. Under proteostasis conditions, the molecular chaperone binding immunoglobulin protein (BiP; also known as GRP78) interacts with three ERS sensors (ATF6, IRE1α, PERK) and maintains them in an inactive state [47]. GSEA further unveiled potential signaling pathways in which the nine ERS-related lncRNAs were associated with upregulated extracellular matrix (ECM)-related processes in the high-risk group. Prior research has indicated that during tumor initiation and progression, the ECM undergoes a remodeling process [48, 49]. This restructured ECM can generate a favorable microenvironment for tumor growth, ultimately promoting tumor cell proliferation, invasion, and metastasis [50, 51]. The pertinent features of ECM remodeling may serve as crucial indicators for tumor clinical staging, early diagnosis, and prognostic evaluation. Meanwhile, lncRNAs hold the capacity to influence the composition of the tumor microenvironment and actively engage in immune regulation by modulating the metabolic processes inherent to tumor cells [52]. As an illustrative instance, lncRNA MALAT1 has been documented to exert its influence by instigating alterations in tumor cell metabolism, specifically within the glycometabolic pathways. These metabolic shifts culminate in the creation of an immunosuppressive microenvironment within the tumor milieu. Consequently, MALAT1 is anticipated to function as a negative regulator of antitumor immunity [53, 54]. NEAT can indeed exhibit an immunoregulatory role in the context of cancer. Notably, tumor specimens characterized by elevated infiltration of cytotoxic CD8 + T cells tend to display diminished levels of NEAT1 expression. Furthermore, NEAT1 contributes to the promotion of tumor growth by hampering cytotoxic T cell-mediated immunity, primarily through the downregulation of the stimulator of interferon genes, cyclic GMP-AMP synthase [55]. We also found pathways related to metabolism in functional analysis, and the mechanism of 9 lncRNA in gastric cancer immune regulation needs to be further studied.

Based on the identified gene mutations within the two distinct risk subsets, we embarked on a comprehensive exploration of the impact of immunotherapy in this context. Our investigation revealed notable disparities in mutation profiles between these two sets, with a heightened prevalence of mutations in genes such as TTN, TP53, and MUC16 within the high-risk subset, as compared to their low-risk counterparts. Notably, TTN emerged as the most frequently mutated gene across the STAD cohort, exhibiting the strongest correlation with TMB [56]. Building upon this observation, it is worth highlighting that prior research has unequivocally established the significance of TTN and MUC16 gene mutations in shaping the prognosis of gastric cancer, while also serving as pivotal biomarkers for guiding the administration of ICIs [57]. Furthermore, compelling evidence from a separate study underscores the intimate association between TP53 mutations and the intricate landscape of tumor immunity. Consequently, the TP53 mutation status emerges as a promising and effective biomarker, holding the potential to predict the response to cancer immunotherapy across diverse cancer types [58].

TMB-H has emerged as a prime candidate biomarker for identifying cancer patients who may derive benefits from immune checkpoint blockade (ICB) therapy. This is based on the underlying premise that increased numbers of mutant proteins can generate antigenic peptides, thereby enhancing immunogenicity [59]. Our study demonstrated that the mutation rate of the TTN gene was the highest in high-risk patients, albeit lower than that observed in low-risk patients. A previous investigation has corroborated the association between increased gastric cancer aggressiveness and a reduced frequency of TTN mutations [60]. This finding further elucidates the poorer prognosis observed in the high-risk group from the perspective of TMB.

Immune-infiltrating cells within the TIME have garnered significant attention due to their pivotal role in tumor progression, exerting a profound impact on the clinical outcomes of cancer patients. A heightened presence of activated memory CD4 T cells within the TIME has been associated with the facilitation of robust immune responses, thereby substantially enhancing the overall prognosis for individuals afflicted by cancer [61, 62]. Follicular helper T cells have been documented as integral contributors to the intricate process of information transmission during B cell differentiation. These specialized T cells play a pivotal role in orchestrating the activation of B cells, facilitating the formation of germinal centers, mediating class conversion of immunoglobulins, and sustaining a durable humoral immune response over the long term [63]. Immature dendritic cells (iDCs) engage in interactions with both T and B cells, contributing to the immune response exclusively upon their activation into mature dendritic cells [64]. Regulatory T cells (Tregs) have garnered considerable attention in the context of tumor immunology. The accumulation of a substantial Treg population within the tumor microenvironment has been consistently linked to an unfavorable prognosis in various cancer types, including but not limited to breast, ovarian, and hepatocellular cancers. However, in instances characterized by chronic inflammation, such as colorectal cancer, the presence of Tregs exerting suppressive effects on the persistent inflammatory milieu proves to be advantageous. This phenomenon is intricately associated with a more favorable clinical prognosis [65]. In reaction to inflammatory stimuli, a substantial influx of neutrophils is mobilized within the TME, subsequently undergoing differentiation into tumor-associated neutrophils (TANs). It is noteworthy that TANs do not represent terminally differentiated immune effector cells; instead, their functional polarization occurs along a spectrum, primarily into N1 (characterized by an anti-tumorigenic phenotype) and N2 (characterized by a pro-tumorigenic phenotype). These dynamic transitions are subject to intricate regulation within the complex milieu of the tumor microenvironment [66]. N1 neutrophils exert a potent inhibitory effect on tumor growth through the production of ROS and TNF-α, concomitant with the downregulation of arginase expression. Moreover, they play a pivotal role in orchestrating the recruitment and activation of immune cells by secreting an array of chemokines. Conversely, N2 neutrophils assume a contrasting role by fostering tumor cell proliferation, migration, and angiogenesis. These effects are mediated by the release of matrix metalloproteinases, vascular endothelial growth factor and enzymes expressing arginase activity [67]. B cells serve as proficient antigen-presenting cells, proficiently eliciting cytotoxic T lymphocyte activity and releasing cytokines that have been implicated in facilitating cancer metastasis. Additionally, B cells possess the capacity to directly eliminate tumor cells through the secretion of the cytotoxic enzyme, granzyme B [68, 69]. In addition to their innate ability to eliminate tumor cells in the absence of prior sensitization, NK cells exert influence over the functionality of other immune cell populations by virtue of their ability to secrete proinflammatory cytokines and chemokines, notably including IFN-γ [70]. CD8 + T cells are recognized for their role in eradicating cancer cells, disrupting immune tolerance, and amplifying the efficacy of immunotherapeutic interventions by targeting the PD-1/PD-L1 immunosuppressive axis [71].

Our immune infiltration analysis revealed a higher overall immune infiltration score in high-risk cohorts compared to low-risk cohorts. A notable increase was observed in the macrophage immune infiltration score within the high-risk group, presenting a significant divergence. Macrophages, highly plastic immune cells, play integral roles in tissue homeostasis, immune response, and inflammation [72]. In a streamlined perspective, macrophage polarization states are classified as either pro-inflammatory M1 (classically activated) or anti-inflammatory M2 (alternatively activated), a classification that echoes the Th1/Th2 polarization of T cells [73]. Pathological conditions with compromised nutrient availability, such as infection, chronic inflammation, metabolic/nutrient imbalance diseases (diabetes, obesity, atherosclerosis), or ischemia/reperfusion events associated with organ transplantation or surgery, instigate metabolic stress that potentially alters macrophage functions, inducing maladaptive polarization states [74–76]. For instance, hypoxic (oxygen-limiting) conditions associated with inflammation or ischemia activate cellular oxygen sensors and the hypoxia-inducible factor (HIF), thereby inducing a metabolic switch from oxidative to glycolytic metabolism and pro-inflammatory polarization, thereby exacerbating the inflammatory response [77, 78]. This hypoxic environment also closely associates with an endoplasmic reticulum stress response. Hypoxia-driven accumulation of tumor-associated macrophages (TAMs) has been linked to unfavorable outcomes [79, 80]. TAMs constitute a polarized M2 macrophage population [81]. The polarized functions of TAMs integrate them into inflammatory mechanisms that promote tumor growth and progression [82]. Additionally, Within the tumor microenvironment, Tregs play a role in regulating homeostasis and facilitating tumor immune evasion. An increase in Treg cells in the tumor immune microenvironment is indicative of a poor prognosis [83]. Enrichment of Tregs inhibits CD4 + T cells, CD8 + T cells, antigen-presenting cells (APCs), monocytes, and macrophages, allowing tumor cells to proliferate more rapidly under immunosuppressive conditions [83]. To explore the relationship among tumor subtypes, we divided STAD patients into two subgroups. Cluster 1, predominantly composed of low-risk patients, exhibited the highest immune scores and the most extensive immune cell infiltration, reflecting the most robust immune function in this cluster. Among the two subgroups, Cluster 1 displayed the greatest degree of CD8 + T cell infiltration and elevated expression of numerous immune checkpoint molecules. Utilizing ssGSEA, we investigated the immune status of different groups, revealing that Cluster 1 had more extensive immune cell infiltration and stronger anti-tumor immunity. Previous studies have demonstrated that the intrinsic ERS responses of cancer cells can influence malignant progression by altering the function of immune cells co-existing within the tumor microenvironment. The impact of immune functions, such as T cell co-inhibition, T cell co-stimulation, type II IFN responses, on tumor cell survival warrants further investigation. Consequently, we hypothesize that the poor prognosis observed in Cluster 2 patients may be attributed to reduced immune cell infiltration and diminished anti-tumor immune function.

Tumor therapy remains a critical area of focus. By screening and analyzing the half-maximal inhibitory concentration (IC50) of potential drugs, we identified medications with proven efficacy against gastric cancer. Our analysis suggests that low-risk patients may be sensitive to oxaliplatin and epirubicin. A study involving 503 patients with locally advanced, resectable esophagogastric adenocarcinoma treated with either three cycles of epirubicin, cisplatin, and fluorouracil (ECF) administered before and after surgery or surgery alone demonstrated that the chemotherapy arm exhibited a significant improvement in overall survival (5-year survival rates, 36% vs 23%) compared with surgery alone [84–86]. In locally advanced, resectable gastric or gastroesophageal junction adenocarcinoma, perioperative fluorouracil plus leucovorin, oxaliplatin, and docetaxel (FLOT) improved overall survival compared with perioperative ECF/ECX [87]. Based on the ERS-related lncRNA prognostic signature, we found that low-risk patients are sensitive to the majority of immunotherapy drugs and may experience enhanced efficacy. We hypothesize that low-risk patients are more likely to elicit anti-tumor immune responses and benefit from immunotherapy. In summary, our ERS-related lncRNA prognostic signature can serve as a novel indicator for evaluating the applicability of immune checkpoint inhibitors (ICIs). Furthermore, in conjunction with drug sensitivity and immune efficacy analyses, we predict that low-risk patients will benefit more from the combination of chemotherapy, targeted therapy, and immunotherapy, providing a foundation for individualized treatment in STAD patients. Additionally, more drugs merit consideration and development for low-risk patients.

Undoubtedly, our study has several limitations. First, the prognostic signature is based on TCGA public database, lacking validation from samples in other databases. Second, while our study confirms that the prognostic signature possesses robust predictive value, in vitro experiments are still required to elucidate the mechanism of ERS in STAD. Third, the association between prognostic features and the effects of targeted therapy, chemotherapy, and immunotherapy in STAD patients necessitates urgent investigation through an extensive number of clinical trials.

In this study, we established an ERS-related lncRNA prognostic signature and delineated its associations with TMB, immune response, and clinical interventions (targeted therapy, chemotherapy, and immunotherapy). This information can serve as a valuable reference for the individualized and precision treatment of STAD.

### Supplementary Information

**Additional file 1.** The List of 330 ERS‑related genes.

**Additional file 2.** The List of 364 differentially expressed ERS‑related genes in the high- and low-risk groups.

**Additional file 3.** The List of drug sensitivity analysis in high- and low-risk groups.

## Data Availability

Major data generated or analysed during this study are available in the TCGA (https://portal.gdc.cancer.gov/) and the GEO database (https://www.ncbi.nlm.nih.gov/geo/). 330 ERS-related genes are included in the article/Supplementary Material. 29 immune signatures (16 immune cells and 13 immune functions) and 47 immune checkpoint genes are available from Additional file 1 of the article entitled “Classification of triple-negative breast cancers based on Immunogenomic profiling” (https://jeccr.biomedcentral.com/). Further inquiries can be directed to the corresponding authors.

## Abbreviations

AUC: Area under the curve
BiP: Binding immunoglobulin protein
CDF: Cumulative distribution function
DEG: Differentially expressed gene
EBV: Epstein-Barr virus
ECM: Extracellular matrix
EMT: Epithelial-mesenchymal transition
ERS: Endoplasmic reticulum stress
FC: Fold change
FDR: False discovery rate
GC: Gastric cancer
GDSC: Genomics of drug sensitivity in cancer
GO: Gene ontology
GSEA: Gene set enrichment analysis
HCC: Hepatocellular carcinoma
HER2: Human epidermal growth factor receptor 2
HIF: Hypoxia-inducible factor
ICB: Immune checkpoint blockade
ICI: Immune checkpoint inhibitor
IC50: Half-maximal inhibitory concentration
KEGG: Kyoto encyclopedia of genes and genomes
LASSO: Least absolute shrinkage, and selection operator
lncRNA: Long non-coding RNA
MSI: Microsatellite instability
MSigDB: Molecular signature database
OS: Overall survival
PD-L1: Programmed cell death ligand 1
ROC: Receiver operating characteristic
STAD: Stomach adenocarcinoma
TAM: Tumor-associated macrophage
TCGA: The cancer genome atlas
TMB: Tumor mutational burden
TIME: Tumor immune microenvironment
t-SNE: T-distributed stochastic neighbor embedding
UPR: Unfolded protein response
